# Effect of Metformin on Myocardial Injury Induced by Hepatic Ischemia-Reperfusion in Rats

**DOI:** 10.3389/fphar.2022.822743

**Published:** 2022-04-01

**Authors:** Wen An, Ju-Seop Kang

**Affiliations:** Department of Pharmacology and Clinical Pharmacology Lab, College of Medicine, Hanyang University, Seoul, South Korea

**Keywords:** hepatic ischemia-reperfusion, oxidative stress, metformin, hepatic injury, myocardial injury

## Abstract

**Background:** There is no effective medication for treatment or prevention of hepatic ischemia-reperfusion (HIR) injury caused by liver transplantation and hepatectomy. This study aimed to investigate the therapeutic effects of metformin on HIR injury and related myocardial injury in rats.

**Methods:** Wistar male rats were randomly divided into four groups: sham group, ischemia-reperfusion group, and IR group treated with metformin 150 mg/kg and 100 mg/kg. Wistar male rats were administered metformin 150 mg/kg, 100 mg/kg or saline 30 min pre-operative and underwent 15 min ischemia and 6 h reperfusion (*n* = 4).

**Results:** Metformin significantly alleviates the injury caused by HIR. Administration of metformin resulted in a significant reduction in the serum levels of alanine transaminase and aspartate transaminase and the activity of malondialdehyde, creatine kinase-MB, and lactate dehydrogenase but maintained high catalase and superoxide dismutase activity. Metformin significantly inhibited the IR-induced elevation of tumor necrosis factor-*α* in liver and heart tissue.

**Conclusion:** Metformin can alleviate hepatic and myocardial injury induced by IR by inhibiting oxidative stress.

## Introduction

At present, the most common and effective treatment for advanced liver diseases is liver resection and transplantation. However, hepatic ischemia-reperfusion (HIR) injury is an inevitable complication of this operation. HIR injury can increase the rate of acute and chronic rejection and leads to 10% early organ failure after liver transplantation, which seriously hinder application and therapeutic effect of liver transplantation (Rampes and Ma, 2019) There is an urgent need to alleviate the injury induced by HIR to improve the survival rate and prognosis of patients after liver transplantation.

The process of HIR injury is complex and includes three phases: the ischemia stage, early reperfusion stage, and late reperfusion stage. The primary damage is caused by rapid ATP consumption induced by anaerobic digestion in the liver, increasing intracellular Ca_2_^+^ level and causing mitochondrial dysfunction during the ischemia stage (Wei-Nan Li, 2015). The acute injury phase, also known as the early reperfusion stage, is characterized by liver injury within 1–6 h of reperfusion and is associated with excessive reactive oxygen species (ROS) production, Kupffer cell activation, and release of pro-inflammatory cytokines such as TNF-α and IL-6 (Crockett et al., 2006). Further progression results in parenchymal dysfunction, direct cellular damage (necrosis, membrane disruptions), and indirect damage through an inflammatory response. Finally, neutrophil infiltration is characterized in the late reperfusion stage and results in ROS production and liver injury (Zabala et al., 2019). The ROS generated during reperfusion flow from the hepatic vein to the right atrium. Therefore, the heart is the first organ receiving the blood flow after HIR and is susceptible to damage. Myocardial injury can be induced by HIR (Nastos et al., 2014).

Patients with type 2 diabetes, especially obese diabetes, should be treated first with metformin. At present, there are many studies on metformin in various fields, including HIR. Monika et al. indicated that metformin reduced ROS production in complex I, increased antioxidant enzyme activity, and reduced postischemia inflammation in fatty liver (Cahova et al., 2015). Li et al. discovered that the signal pathway TLR4/NF-κB associated with metformin attenuates IR injury of fatty liver (Li et al., 2020). Metformin can achieve anti-inflammatory and antioxidant effects by activating AMPK and different signals downstream, reducing the release of inflammatory factors and ROS production (Dehkordi et al., 2018). Therefore, metformin plays a protective role in HIR injury, mainly through antioxidation. It has been reported that metformin could regulate TIGAR inhibit glycolysis then regulate Th17/Treg balance, inhibit the release of liver inflammatory factors, and finally play a role in inhibiting the occurrence of liver injury caused by ischemia-reperfusion (Jiang et al., 2021) Metformin can also induce cardiac protection and improve cardiac function (R. J. Legtenberg 2002) in rats with myocardial ischemia-reperfusion injury and myocardial infarction by activating AMP activated protein kinase (AMPK) pathway (Solskov et al., 2008) and AMPK-eNOS signaling pathway (Jo et al., 2020), respectively. Moreover, metformin not only induces protective effect in rats, but also has corresponding cardiac protective effect in humans (El Messaoudi et al., 2015)

The primary purpose of this study is to investigate the therapeutic effect of metformin on HIR and myocardial injury during the early reperfusion stage by establishing a 15 min hepatic ischemia model.

## Materials and Methods

### Animals

Male Wistar rats (250–300 g) were obtained from Orient Bio (Gyeonggi-do, Korea). All rats were housed in a temperature-controlled environment (23°C) with two rats per cage on a 12 h light/dark cycle, with free access to food and water. 1 week was allocated for animal acclimation to the new housing. Animal protocols were approved by the Institutional Animal Care and Use Committee of Hanyang University (IACUC approval number: 2010-0113A). We estimate the sample size of animal experiments according to the “resource equation” method (E value) and the 3R (replacement, reduction, refinement) principle. E = (4 * 4) - 4 = 12. E value is 12 > 10, and <20, which is the acceptable limit and hence can be considered as adequate sample size. (Charan and Kantharia, 2013)

### Induction of Ischemia-Reperfusion Injury

**FIGURE 1:**
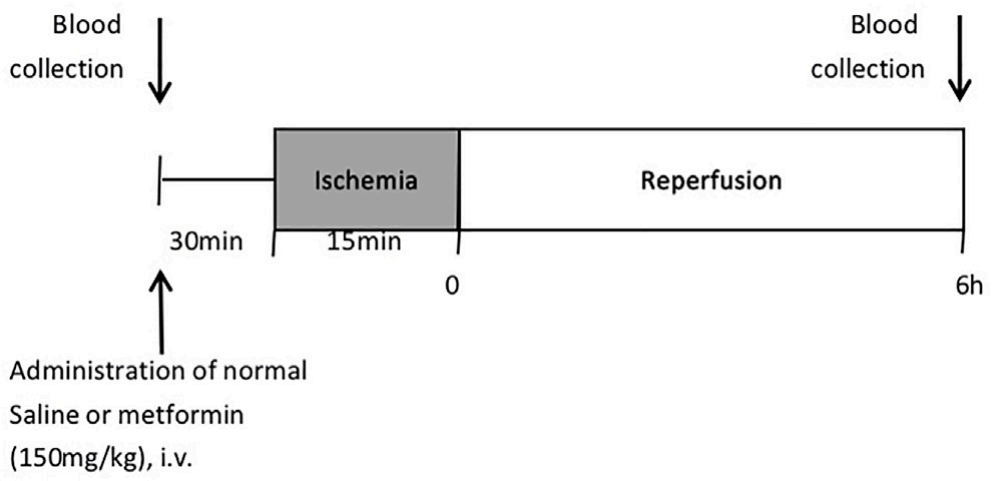
Experimental design.

The overall experimental protocol of the present study is described in Figure 1. Male Wistar rats of approximately 9 weeks old were randomly divided into four groups (*n* = 4): the sham group, the ischemia-reperfusion (IR) group, IR model treated with metformin-high (150 mg/kg), and IR model treated with metformin-low (100 mg/kg). All rats were fasted for about 12 h before the operation. Rats were anesthetized with isoflurane by inhalation (Priamal, United States) and subjected to 15 min hepatic ischemia by portal vein ligation and 6 h after reperfusion. Blood was collected from the tail vein before injection of metformin and the heart after 6 h of reperfusion.

After blood collection, the IR group was administered 0.3 ml of normal saline, and the experimental groups were administered metformin by caudal vein. At 30 min after injection of metformin, under respiratory anesthesia, a ventral incision was made in the abdomen. After ischemia, a change in the color of the liver surface was seen 5 min later, the abdominal incision was temporarily closed with a surgical clamp, and the abdominal wounds were sutured after reperfusion. When the animals recovered from anesthesia, they were placed back into the cage and allowed access to water ad libitum. The body temperature of the rats was maintained at 37°C through a warm pad during the ischemia stage. Sham-operated rats underwent identical surgical procedures except for ischemia. The rats were anesthetized with isoflurane by inhalation 6 h after reperfusion and died after blood collection and organ tissue collection (Hirakawa et al., 2019)

### Metformin Administration

Metformin was purchased from Tokyo Chemical Industry (Tokyo, Japan) and was dissolved in sterile saline. Doses were administered by intravenous injection. The dosage and injection method refer to the previously reported papers. (Cahova et al., 2015; Palee et al., 2020)

### Blood, Serum, and Tissue Sampling

At the end of the reperfusion period (6 h), the animals were anesthetized to collect blood, liver, and heart tissue. Blood sampling was collected immediately from the heart and allowed to clot at room temperature for 30 min. Then, the serum was separated using a centrifuge at 4,000 rpm for 10 min at 4°C and stored at −80°C until use. Heart and liver were removed, rinsed thoroughly with saline, and divided into two sections. The first section was placed in 10% phosphate-buffered formalin at room temperature for histopathological examination, while the other section was immersed immediately in liquid nitrogen and stored at −80°C.

### Evaluation of Liver Function

Serum levels of alanine transaminase (Boeddinghaus et al., 2020) and aspartate transaminase (AST) were measured using a Hitachi clinical analyzer 7,180 (Hitachi Ltd., Tokyo, Japan).

### Determination of Myocardial Injury Biomarkers

Lactate dehydrogenase (LDH) activity was determined via a commercial LDH assay kit (Abcam, ab102516, United Kingdom). NADH standard and supernatants (50 μl) separated at 4°C at 10,000 × *g* for 15 min were transferred into a 96-well plate for LDH detection, and a mixed detection reagent (50 μl) was added to each well. The absorbance (450 nm) was measured on a micro-plate reader every 3 min for 30 min at 37°C. Creatine kinase MB (CK-MB) isoenzyme was measured by an enzyme-linked immunosorbent assay (Bhoori et al., 2020) technique using a kit supplied by Novus Biologicals (NBP2-75313, United States).

### Measurement of Malondialdehyde Levels in the Liver and Heart

Lipid peroxidation was estimated by measuring malondialdehyde (MDA) content, a product of lipid oxidation. Liver and heart tissues were homogenized with RIPA buffer to obtain a 10% homogenate (50 mg of tissue in 0.5 ml of cold buffer) using a homogenizer. The MDA level was detected using a kit purchased from Cayman (MI, United States). The assay depends on the MDA-thiobarbituric acid (TBA) adduct, which is formed by reaction of MDA in samples with TBA under high temperatures (90–100°C) and acidic conditions and can be measured calorimetrically at 532 nm.

### Measurement of Superoxide Dismutase Activity in the Liver

Superoxide dismutase (SOD) was assessed using a kit purchased from Cayman. The assay uses a tetrazolium salt for detection of superoxide radicals generated by xanthine oxidase and hypoxanthine. The kit mainly measured Mn-SOD in mitochondria, although it also included cytosolic Cu/Zn SOD and extracellular SOD.

### Measurement of Catalase Activity in the Liver and Heart

Catalase (CAT) was assessed using a kit purchased from Cayman. The assay is based on the formaldehyde produced by the enzyme’s reaction with methanol in the presence of an optimal concentration of H_2_O_2_ and is measured calorimetrically at 540 nm.

### Histopathological Evaluation of Liver and Heart Tissues

Liver and heart tissues were stored in 10% formalin before being fixed in paraffin. The formalin-fixed liver and heart samples were embedded in paraffin blocks and cut into sections of 5 μm thickness. The sections were stained with hematoxylin and eosin (H&E) as in the standard protocol.

### Western Blot Analysis

A western blot of liver and heart homogenate was performed to confirm the expression of TNF-α. Frozen liver and heart samples were dissolved in lysis buffer (Cayman, 10010263). The homogenates were incubated on ice for 30 min, followed by centrifugation at 4°C at 12,000 rpm for 5 min. The Bradford protein assay determines the protein concentration of the supernatant with BSA (Bovine albumin) as a standard. Equal amounts of proteins (20 g) from each sample were separated using 12% sodium dodecyl sulfate-polyacrylamide gel electrophoresis, and the proteins were transferred to nitrocellulose membranes. To reduce non-specific binding, the membranes were blocked with 2.5% non-fat milk in 1×TBST buffer (Tris-Hcl, NaCl, tween20) before being probed with anti-TNF-α antibody (Santa Cruz, sc-52746, 1:500 dilution, United States) and *β*-actin (Santa Cruz, sc-47778, United States) overnight at 4°C. After being washed with 1×TBST buffer three times each for 10 min, the membranes were incubated with peroxidase-conjugated secondary antibody (Goat anti-Mouse IgG, 1:2000, Invitrogen, United States) at room temperature for 1 h and washed with 1×TBST buffer three times each for 10 min. Blots were visualized using the developing solution Dyne ECL star (Dyne Bio, United States). Staining images were captured with a ChemiDoc XRS (Bio-Rad, United States). The band density was analyzed using ImageJ software (v8., United States).

### Statistical Analysis

All data were expressed as mean ± standard deviation (SD). Statistical analysis was performed using one-way analysis of variance (ANOVA) followed by Tukey’s multiple tests using GraphPad Prism software v.8 (GraphPad Software Inc., La Jolla, CA, United States). A significant difference was assumed for values of *p* < 0.05.

## Results

### Effects of Metformin on Serum ALT and AST

**FIGURE 2:**
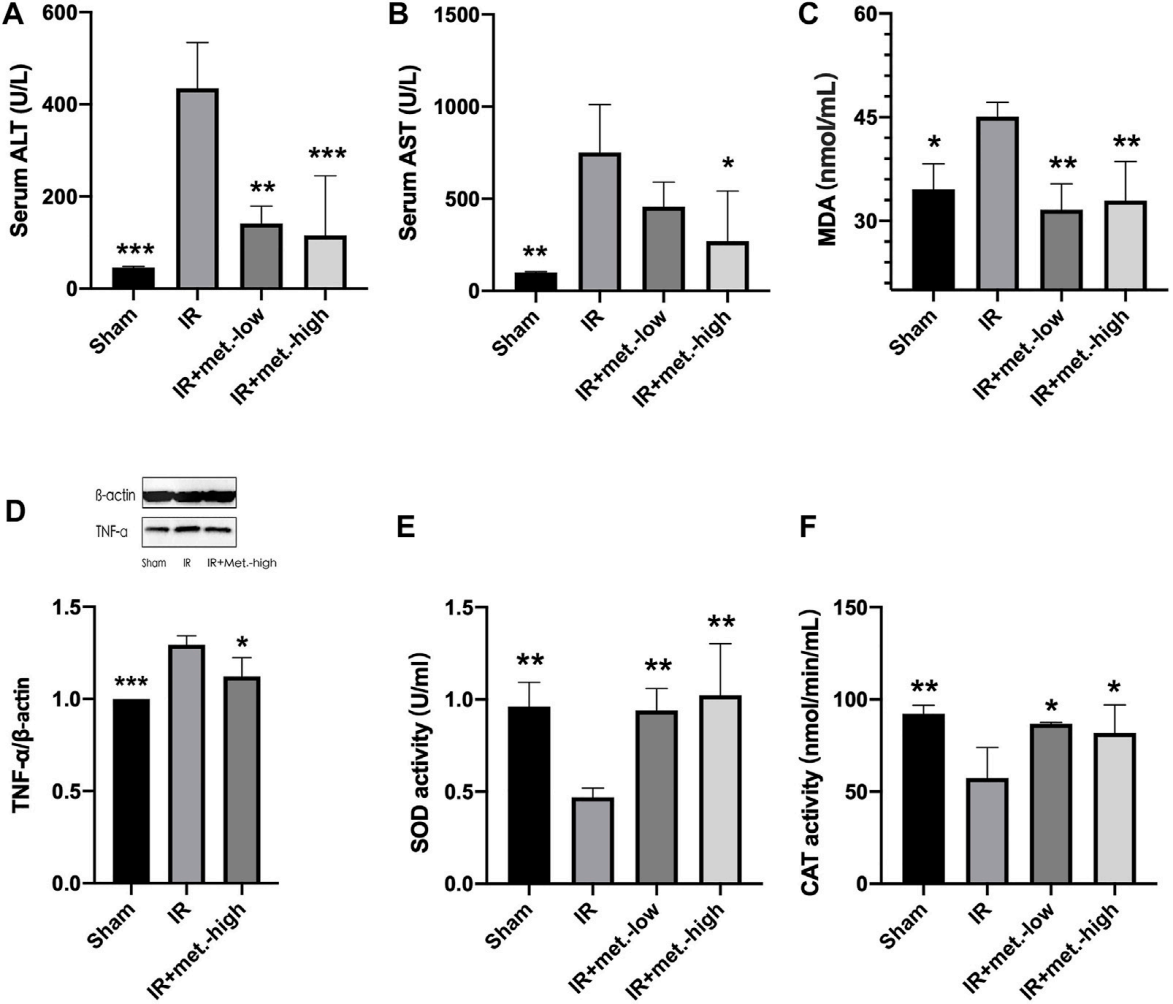
Changes in liver tissue. Serum samples were collected after 6 h reperfusion for measuring **(A)** ALT and **(B)** AST. Tissue samples were collected after 6 h reperfusion for measuring **(C)** MDA, **(D)** TNF-α, **(E)** SOD, and **(F)** CAT. Data are presented as mean ± SD (*n* = 4) and compared by one-way ANOVA. *p < 0.05, **p < 0.01, ***p < 0.005, ****p < 0.001 vs. IR. IR + met.-low is rats underwent IR surgery and were treated with metformin-low 100 mg/kg. IR + met.-high is rats underwent IR surgery and were treated with metformin-high 150 mg/kg.

At 6 h after reperfusion, the mean value of ALT in the IR group was significantly increased compared to that in the sham group (*p* < 0.005), and the mean value of the IR group treated with metformin-high 150 mg/kg (*p* < 0.005) and metformin-low 100 mg/kg (*p* < 0.01) were significantly decreased compared to the untreated IR group. (Figure 2A) Meanwhile, after reperfusion 6 h, the mean value of AST in the IR group was significantly increased compared to that in the sham group (*p* < 0.01), and the mean value of the IR group treated with metformin-high 150 mg/kg (*p* < 0.05) was significantly decreased compared to the untreated IR group. There was no significant difference between metformin-low 100 mg/kg (*p* < 0.01) and IR group. (Figure 2B)

### Metformin Improves Liver Function Following IR Injury

At 6 h after reperfusion, the mean values of MDA (*p* < 0.05) in the IR group were significantly increased compared to those in the sham group, and the mean values of the IR group treated with metformin-high 150 mg/kg (*p* < 0.05) and metformin-low 100 mg/kg (*p* < 0.05) were significantly decreased compared to those in the untreated IR group. (Figure 2C) The trend of TNF-α was the same as that of MDA, but there was no statistical significance between he IR group treated with methformin-low 100 mg/kg group and IR group, so it was not shown in the results. (Figure 2D) Meanwhile, 6 h after reperfusion, the mean SOD activity in the IR group was significantly decreased compared to that in the sham group (*p* < 0.05), and the mean values in the IR group treated with metformin-high150 mg/kg (*p* < 0.05) and metformin-low100 mg/kg (*p* < 0.05) were significantly increased compared to those in the IR group. (Figure 2E) CAT activity in IR (*p* < 0.001), IR treated with metformin-high 150 mg/kg (*p* < 0.01), and metformin-low 100 mg/kg (*p* < 0.01) groups showed almost similar to SOD. (Figure 2F)

### Effect of Myocardial Injury on Biomarkers

**FIGURE 3:**
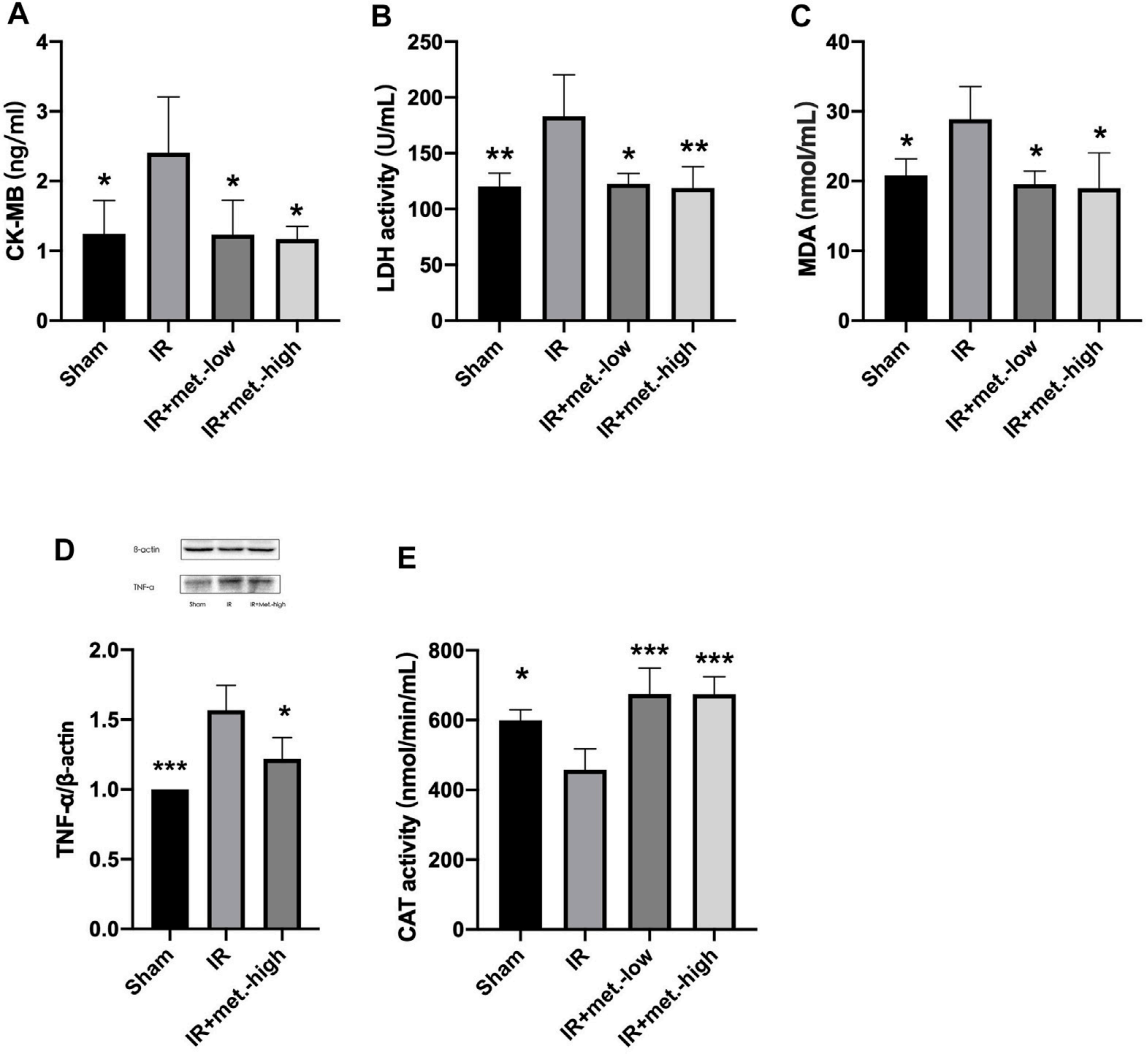
Changes in heart tissue. Serum samples were collected after 6 h reperfusion for measuring **(A)** CK-MB and **(B)** LDH. Tissue samples were collected after 6 h reperfusion for measuring **(C)** MDA, **(D)** TNF-α, and **(E)** CAT. Data are presented as mean ± SD (*n* = 4) and compared by one-way ANOVA. *p < 0.05, **p < 0.01, ***p < 0.005, ****p < 0.001 vs. IR. IR + met.-low is rats underwent IR surgery and were treated with metformin-low 100 mg/kg. IR + met.-high is rats underwent IR surgery and were treated with metformin-high 150 mg/kg.

At 6 h after reperfusion, the mean value of CK-MB in the IR group was significantly increased compared to that in the sham group (*p* < 0.05), and the mean value of the IR treated with metformin-high 150 mg/kg (*p* < 0.05), and metformin-low 100 mg/kg (*p* < 0.05) groups were significantly decreased compared to that of the IR group. (Figure 3A) After 6 h of reperfusion, LDH activity in the IR (*p* < 0.05) and IR treated with metformin-high 150 mg/kg (*p* < 0.01), and metformin-low 100 mg/kg (*p* < 0.01) groups showed similar results to those of CK-MB. (Figure 3B)

### Metformin Improves Heart Function After IR Injury

At 6 h after reperfusion, the mean values of MDA (*p* < 0.05) in the IR group were significantly increased compared to those in the sham group, and the mean values of the IR group treated with metformin-high 150 mg/kg (*p* < 0.05), and metformin-low 100 mg/kg (*p* < 0.05) groups were significantly decreased compared to those in the IR group. (Figure 3C) TNF-α was the same as that of MDA, but there was no statistical significance between he IR group treated with methformin-low 100 mg/kg group and IR group, so it was not shown in the results. (Figure 3D) Meanwhile, after reperfusion 6 h, the mean value of CAT activity in the IR group was significantly decreased compared to the sham group (*p* < 0.05), and the mean values of IR treated with metformin-high 150 mg/kg (*p* < 0.005) and metformin-low 100 mg/kg (*p* < 0.005) groups were significantly increased compared to the IR group (Figure 3E)

### Histopathological Examination of Liver and Heart Tissues

**FIGURE 4:**
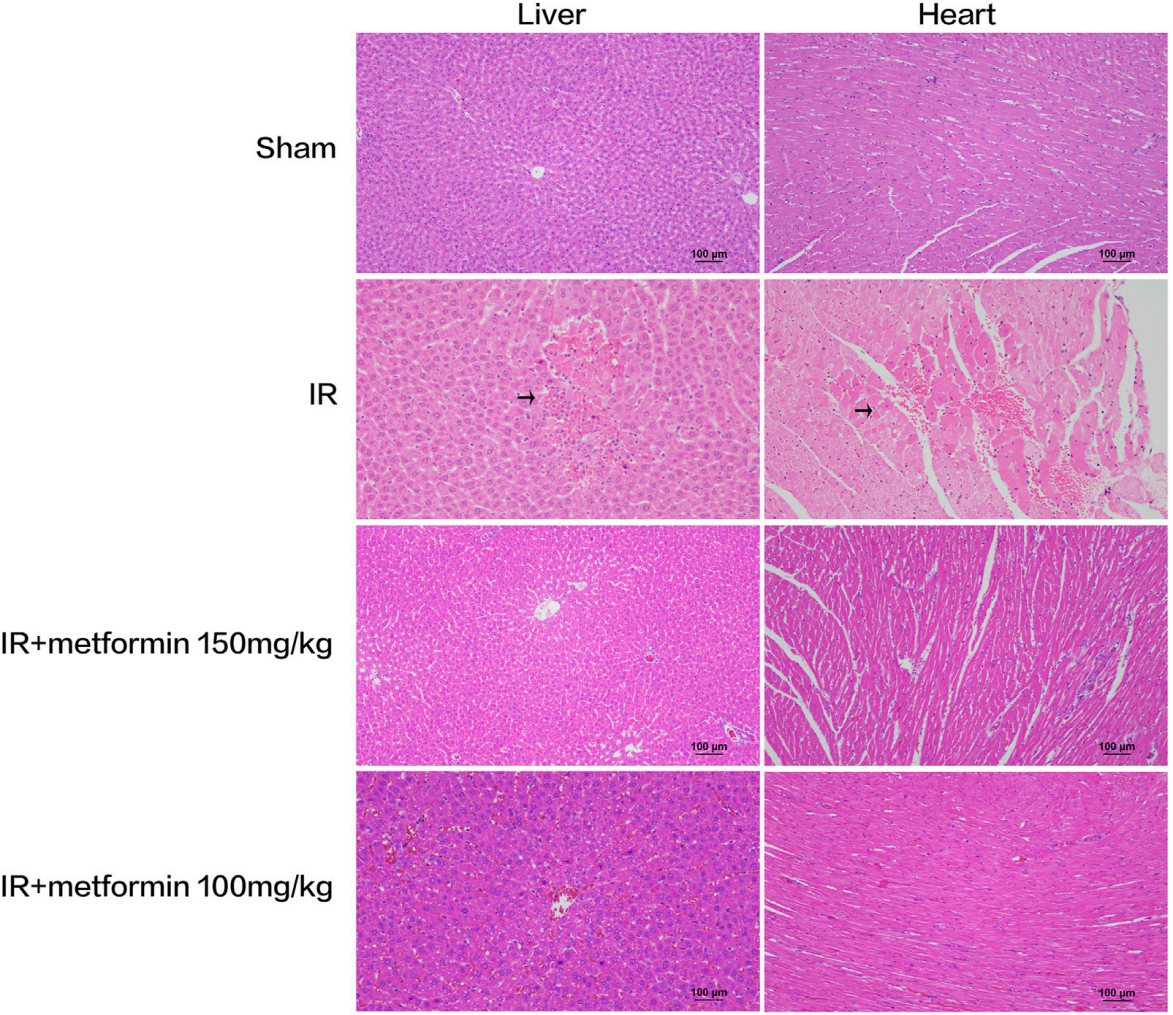
Histopathological examination of liver and heart tissue at 6 h after ischemia-reperfusion (IR). There were obvious necrosis of hepatocytes and cardiomyocytes in IR group (arrows). At the same time, the liver tissue treated with metformin 100 mg/kg showed slight steatosis. (Scale bar = 100 μm).

Liver and heart histopathology was assessed by congestion, necrosis, hemorrhage, and oedema. There was no damage to hepatocytes and cardiomyocytes in saline-treated sham rats. Depend on liver histopathology results, the ischemic liver’s reperfusion resulted in hepatocyte necrosis, congestion, hemorrhage, and oedema in the IR group. In treated with metformin-high 150 mg/kg group, there was no significant change in hepatocyte status. However, that showed slight steatosis in treated with metformin-low 100 mg/kg group. On the other hand, according to the results of cardiac histopathology, myocardial cells in the IR group showed necrosis, congestion, hemorrhage, and oedema. Simultaneously, the state of cardiomyocytes tended to the sham operation group in treated with 150 mg/kg and 100 mg/kg of metformin. (Figure 4) A scoring of histopathological findings is summarized in Table 1.

**TABLE 1:** Histopathology scoring of liver and heart tissue at 6 h after ischemia/reperfusion (IR) procedure. IR, ischemia/reperfusion only group; IR + met-high, rats underwent IR procedure and treated metformin (150 mg/kg); IR + met-low, rats underwent IR procedure and treated metformin (100 mg/kg); Deg. And Nec., degeneration and necrosis; Cong., congestion; Ed., edema; Hg., hemorrhage (−) nil, (+) mild (++) nil to mild, (+++) severe, (++++) more severe.

Groups	Liver				Heart			
	Deg. and Nec	Cong	Ed	Hg	Deg. and Nec	Cong	Ed	Hg
Sham	−	−	−	−	−	−	−	−
IR	++	−	−	+	++	+	+	++
IR + met-high	−	−	−	−	−	−	−	−
IR + met-low	−	−	−	−	−	−	−	−

## Discussion

Many clinical factors affect the prognosis of liver transplantation, including myocardial infarction caused by tissue ischemia, stroke, and thrombosis (Hirakawa et al., 2019). Therefore, this study aimed to evaluate the protective effect of metformin on HIR injury in rats and the related distant cardiac damage.

Ischemia for 15 min followed by reperfusion for 6 h resulted in a significant deterioration of liver function. Compared with sham-operated animals, levels of ALT, AST, and MDA were significantly increased in HIR injured rats. These parameters are widely used to evaluate liver function. In conclusion, these parameters confirmed HIR in our experiments.

In addition, our results showed that the serum CK-MB level and LDH activity in the HIR groups were significantly higher than those in the sham operation group, which confirmed that IR injury to the liver not only affected the liver, but also led to distant myocardial injury. In addition, necrosis, hyperemia, hemorrhage, and edema of myocardial cells were observed by histopathological examination. This finding suggests that HIR rarely occurs in isolation. The experimental results indicated that metformin treatment can significantly inhibit the deterioration of liver function and related myocardial injury. In addition, metformin therapy reduces the structural damage in the liver and heart caused by IR damage. It can be observed from the data that the effect of metformin-high 150 mg/kg is slightly better than metformin-low 100 mg/kg.

Oxidative stress induced by ROS production is a key factor in HIR injury and plays an important role in the progression of this disease. During the ischemia period, the liver produces substantial ROS that cannot be eliminated. The ROS cause lipid peroxidation and damage of the bio-membrane, resulting in an increased level of MDA, the product of lipid peroxidation, leading to apoptosis and liver injury. (Yang et al., 2021), (Perluigi et al., 2012) Under normal physiological conditions, the body constantly produces ROS, which is cleared by antioxidant enzymes (SOD, CAT, etc.) to maintain a dynamic physiological balance. However, ischemia affects ROS homeostasis. Our data show that metformin can upregulate the activity of CAT and SOD, enhance the ability to scavenge excess ROS, and reduce the hepatocyte damage caused by ROS. There is evidence that the antioxidant mechanism of metformin can be divided into two processes: 1) direct capture of hydroxy radicals and 2) activation of antioxidant enzymes such as CAT to decompose H_2_O_2_ (Dehkordi et al., 2018) Our results are consistent with articles published previously (Zeng et al., 2019)

During brain ischemia, the increase and decrease of AMP and ATP levels can activate AMPK. AMPK can stabilize Nrf-2 and induce its gene expression. Induction of the Nrf-2 pathway is related to an increase of antioxidant enzymes such as CAT and SOD (Ashabi et al., 2015). In this regard, we also believe that metformin can stimulate the activation of AMPK and play an antioxidant role in HIR, which needs to be further confirmed.

The inflammatory response plays a critical role in tissue injury induced by HIR. Kupffer cells and endothelial cells are pre-stimulated during hepatic ischemia. In the early stage of reperfusion, Kupffer cells are activated by the complement release of many pro-inflammatory factors (TNF-α, IL-6, TLR4, IL-1, Ccr2, etc.) (Cahova et al., 2015), (Li et al., 2019), (Zhang et al., 2017) and ROS, leading to liver parenchyma injury and recruiting neutrophils, macrophages, and other inflammatory cells to amplify the inflammatory response. This pro-inflammatory activation of liver immune cells is regarded as direct cell injury secondary to ischemic injury (Cahova et al., 2015). TNF-α, as an important mediator of the inflammatory response in HIR injury, not only induces apoptosis of ischemic hepatocytes (Yang et al., 2021), but also causes distal organ damage (Zhang et al., 2017). Apoptosis induced by TNF is due to the combination of TNF and TNFR1 and the formation of an apoptotic signal complex. HO-1 can inhibit the apoptotic signal received by TNF/TNFR1 (Li et al., 2019). TNF-α secretion after IR is regulated by Nox2 and P47phox, which inhibit the production of Nox1-dependent TNF-α by Kupffer cells and Nox2 in the liver (Spencer et al., 2013). Some studies have shown that down-regulating the phosphorylation of AMPK(Ding et al., 2020), the NF-ĸB pathway, and inflammatory molecules can reduce the inflammatory response induced by HIR (Zhang et al., 2017). In addition, previous articles have confirmed that metformin can reduce IR injury of the fatty liver by inhibiting the TLR4/NF-ĸB pathway (Li et al., 2020) and the level of IR injury by inhibiting the AMPK pathway (Dehkordi et al., 2018). In conclusion, we speculate that metformin can achieve anti-inflammatory and antioxidant effects by inhibiting the Nrf-2/HO-1 pathway. The anti-inflammatory effect and the metformin pathway need to be confirmed by subsequent experiments.

Isoflurane precondition for 1.5–6 h can reduce the injury caused by ischemia-reperfusion in liver, brain, myocardium and kidney. (Qin et al., 2014; Ran et al., 2015; Peng et al., 2019; Wang et al., 2021) However, there was no precondition with isoflurane in this study, but the ischemia-reperfusion experiment was carried out immediately after isoflurane anesthesia. Therefore, the influence of isoflurane on the results can be ruled out.

There are some limitations to this study. First, since the number of samples is small, there might be some discrepancies. Secondly, the ischemia time is less than 15 min, which can damage the liver and heart, but the degree of damage is lighter than that after 30 min or 1 h. The reperfusion time is only 6 h, which is in the early stage of IR. Injury is only caused by ROS produced by Kupffer cells. After 6 h, a complex process of ROS production after neutrophil recruitment is involved. Finally, this study lacks echocardiographic measurement and hemodynamic measurement to judge cardiac function, but we compared and summarized the previously reported articles. Palle, S et al. reported that rats with myocardial ischemia-reperfusion injury treated with metformin at all doses (100, 200, 400 mg/kg, i. v.) showed improved SV, lvesp, + dP/dt, % LVEF and RPP. (Palee et al., 2020) Moheimani, H.R el. al also confirmed that metformin could eliminate the attenuation of% LVDP and RPP caused by myocardial ischemia-reperfusion. (Moheimani et al., 2021) John, w et al. showed that metformin significantly increased the ejection fraction after treatment in mice with myocardial ischemia-reperfusion. (Calvert et al., 2008) Woori, Jo et al. obtained the conclusion that metformin can improve the left ventricular diastolic function in rats with myocardial ischemia-reperfusion by analyzing echocardiography. (Jo et al., 2020)

Although many studies have focused on the effect of metformin on HIR injury, the distal organ damage caused by HIR, such as damage to the heart, is often ignored. Our study is mainly aimed at addressing this problem. The results show that metformin not only alleviates liver injury caused by HIR, but also alleviates myocardial injury. The mechanism needs to be further studied.

## Data Availability

The raw data supporting the conclusion of this article will be made available by the authors, without undue reservation.

## Abbreviations

ALT, Alanine transaminase; AST, Aspartate transaminase; LDH, Lactate dehydrogenase; CAT, Catalase; SOD, Superoxide dismutase; CK-MB, Creatine kinase MB; MDA, Malondialdehyde; TNF-α, Tumor necrosis factor-α; IR, Hepatic ischemia-reperfusion; HIR, Hepatic ischemia-reperfusion.
